# Longitudinal effects of basic psychological need support on the development of intrinsic motivation and perceived competence in physical education. A multilevel study

**DOI:** 10.3389/fpsyg.2024.1393966

**Published:** 2024-07-05

**Authors:** Felix Kruse, Sonja Büchel, Christian Brühwiler

**Affiliations:** ^1^Institute of Physical Education, Sports and Health, St. Gallen University of Teacher Education, St. Gallen, Switzerland; ^2^Institute of Education and Professional Studies, St. Gallen University of Teacher Education, St. Gallen, Switzerland; ^3^Vice-President’s Office for Research and Development, St. Gallen University of Teacher Education, St. Gallen, Switzerland

**Keywords:** basic psychological needs, self-determination theory, need support, physical education, MSEM, intrinsic motivation, perceived competence, autonomy support

## Abstract

**Background:**

Grounded in self-determination theory, this article deals with a multidimensional measurement of the support of the basic psychological needs and the individual and combined effects on the development of intrinsic motivation and perceived competence in physical education. In addition to the supportive teacher behaviors of autonomy support, competence support and relatedness support, peer relatedness support is examined as an additional factor.

**Methods:**

A total of 1,047 students from 72 classes from various German-speaking Swiss cantons took part in the study. The hypothesized four factorial structure was analyzed using multilevel confirmatory factor analyses. Longitudinal measurement invariance testing of intrinsic motivation and perceived competence indicates scalar measurement invariance. Multilevel regression analyses were specified to analysis the longitudinal effects on the development of intrinsic motivation and perceived competence, whereby both the effects of the individual factors as well as the adjusted effects under the inclusion of all predictors were examined.

**Results:**

Results of multilevel confirmatory factor analysis indicate that the hypothesized four-factor model (e.g., CFI = 0.97; TLI = 0.96; RMSEA = 0.04; SRMR between = 0.11) is to be favored over alternative models at both levels. Regarding the prediction of the development of intrinsic motivation and perceived competence our study underlines the predictive value of basic psychological need support. The models that examine the effects of the individual predictors indicate that the effects are largely consistent with expectations at both the class and individual level. At class level, however, autonomy support appears to be no significant predictor for the development of intrinsic motivation (*p* < 0.10), but for perceived competence (*p* < 0.05). Peer relatedness support is a significant predictor for both outcome variables at both levels of analysis. Regarding the simultaneous integration of all predictors, only the effects of peer relatedness support remain significant for both outcome variables.

**Discussion:**

The empirical support of the multidimensionality of the instrument is particularly interesting in the context of the common conceptualization of a unidimensional measurement of autonomy support or a composite factor of psychological need support, whereby only few studies have adequately tested the factorial validity. Although significant effects of supportive teacher behaviors can be demonstrated in the multilevel regression analyses, it is also indicated that the different dimensions lack of incremental predictive validity. Particularly noteworthy is the rarely investigated role of peer relatedness support, which has been shown to be a meaningful predictor, even when supportive teacher behaviors are taken into account.

## 1 Introduction

Motivation is one of the dominant topics in the field of sports psychology and it’s contexts like physical education (PE) (Lindahl et al., 2015). This is hardly surprising, as participation and learning success in PE is characterized by a strong motivational component (Chatzisarantis and Hagger, 2009; Ntoumanis and Standage, 2009), which should ultimately also be evident in the context of life-long sports participation (Telama, 2009). PE offers a unique opportunity for children and adolescents to participate in sport and physical activity by not only becoming physically active during school hours, but also gaining the necessary skills and motivation to become physically active outside of school (Taylor et al., 2010; Shen, 2014; Vasconcellos et al., 2020). The relevance is striking, as the already insufficient physical activity decreases from childhood through adolescence (Dumith et al., 2011; Corder et al., 2019), despite being associated with a number of health conditions, such as cardiovascular or metabolic health as well as mental and cognitive health (Janssen and LeBlanc, 2010; Poitras et al., 2016; Biddle et al., 2019; Whooten et al., 2019). Regarding ongoing participation in physical activity into adulthood, the contribution of affective experiences in PE can be emphasized (Ladwig et al., 2018). Enjoyment and the perception of physical competence can be considered as particularly important in this regard as well as regarding the drop out of organized sports in children and youth (Stodden et al., 2008; Crane and Temple, 2015).

Self-determination theory (SDT) has proven to be a potent framework for describing the development and effects of motivation in this context. SDT’s organismic integration theory differentiates between different types of motivation along a continuum from amotivation (a lack of motivation) to intrinsic motivation (e.g., students develop a genuine enjoyment or interest in PE; Ryan and Deci, 2017). For PE, beneficial associations of intrinsic motivation with adaptive outcomes such as physical activity intentions (Vasconcellos et al., 2020) as well as with an increase in physical activity levels (Lonsdale et al., 2019) have been demonstrated. Embedded in SDT, the theory of the three basic psychological needs emphasizes that for the development of high-quality motivation and the best possible functioning of oneself, the basic needs of autonomy, competence and relatedness must be satisfied. Autonomy can be understood as the perception of willingness of one’s own experiences and actions. Competence is defined as the sense of effectiveness and mastery in interacting with the world, whereas relatedness is understood as connectedness with meaningful others, satisfaction with the social world and the feeling of being accepted (Ryan and Deci, 2017). Research could demonstrate that the satisfaction of these basic psychological needs is vital for personal growth, well-being and the development of autonomous motivation (Jang et al., 2009; Vasconcellos et al., 2020). Furthermore, a large number of studies have shown that the satisfaction of these basic psychological needs can be promoted by supportive environments (e.g., Roth et al., 2007; Vasconcellos et al., 2020). As social agents, PE teachers as well as peers have the potential to be supportive factors regarding the satisfaction of basic psychological needs and ultimately intrinsic motivation and well-being during PE class (Cheon and Reeve, 2013; White et al., 2021). Need-supportive teacher behaviors include the support of autonomy in, e.g., respecting student’s attitudes and suggestions, providing meaningful rationale and giving choice (Lonsdale et al., 2019), competence support, which refers to teachers’ provision of guidance on student performance to fosters their perception of competence and the feeling to be capable of successfully carrying out own endeavors (Sanchez-Oliva et al., 2014; Sparks et al., 2017), and relatedness support, which refers to the feeling of being understood by significant others and feeling connected to them. With regard to teachers, relatedness supportive teacher behaviors can involve devoting time to the students, reflecting the emotional needs of the students in the relationship with the teacher, and treating the students in an appreciative and warm manner (e.g., Sparks et al., 2017). Indeed, need supportive teacher behaviors have been shown in a large number of studies to promote, e.g., autonomous motivation, skill development and future intentions to exercise (Cheon et al., 2012; Sanchez-Oliva et al., 2014; Behzadnia et al., 2018).

In addition to teachers, peers can also be identified as important social agents in PE (e.g., Gairns et al., 2015). The term peer relatedness support can be understood as supportive aspects that refer to the satisfaction of relatedness by peers. In contrast to the need supportive behaviors of teachers, peer relatedness support has only been considered in a few studies (Vasconcellos et al., 2020). In addition, teachers and peers have mostly been studied in isolation (Gairns et al., 2015). Despite this limited attention to peer relatedness support, previous studies emphasize that there are associations with greater autonomous motivation and a reduction in anxiety in PE (e.g., Cox et al., 2009, 2011). Teachers and peers presumably have different ways of influencing potential positive outcomes. For example, it can be assumed that autonomy support is largely provided by the teacher, as they are to be understood as a higher authority in the classroom and can therefore influence the autonomy of the students to a greater extent. The same applies to competence support in that the feedback also largely originates from the teacher, so that s/he is able to influence the students’ perceived competence to a greater extent (e.g., Koka, 2014; Vasconcellos et al., 2020). In contrast, relatedness can likely be understood as being stronger influenced by peers, as they interact with each other on a broader scale throughout the day, rather than just during, e.g., PE class (Vasconcellos et al., 2020).

Vasconcellos et al. (2020) were further able to show in their recent meta-analysis that most of the evidence regarding social contexts and constructs of SDT can be found in the area of autonomy support. Almost three times as many studies have examined the teacher’s autonomy support compared to competence or relatedness support. Regardless of the SDT’s theoretical presumption that all psychological needs are deemed essential for an individual (Ryan and Deci, 2017), a unidimensional approach was mostly used when it comes to supportive factors, which focused solely on autonomy support (Su and Reeve, 2011; Sanchez-Oliva et al., 2014). This may be reasonable, as SDT postulates that all psychological needs are promoted by autonomy support (Ryan and Deci, 2017). In this context, however, it must be emphasized that autonomy support is understood in many operationalizations as an integrative term of an overall need supportive teacher behavior, including competence and/or relatedness support. Only few studies have examined need supportive teacher behaviors in PE in the context of a multidimensional approach, encompassing autonomy support, competence support and relatedness support as individual dimensions (Sanchez-Oliva et al., 2014; Ahn et al., 2019).

Therefore, the purpose of the present study is, firstly, to investigate the individual and combined effects of need supportive teacher behaviors on the development of students’ intrinsic motivation and perceived competence and, secondly, to integrate peer relatedness support as an additional dimension. As mentioned, only few studies have investigated the multidimensionality of basic psychological need support in PE. Moreover, only few studies have analyzed need supportive behaviors in a multilevel context, ignoring the nested data structure, which can lead to biased results (Lüdtke et al., 2009; Huang and Cornell, 2016). However, intra-class correlations (ICC1) indicate a substantial amount of variance between classes of 19% of a composite need support scale (Ahn et al., 2021) as well as for relatedness support (Leo et al., 2023), demonstrating that multilevel modeling is indicated. The study by Ahn et al. (2019) is the only example we are aware of that addresses both the multidimensionality of need support and the clustered data structure. Taking up this desideratum, the authors conducted multilevel factor analyses (MEFA, MCFA) with the popular short version of the TASCQ. The frequently used composite or latent scores (e.g., Van Den Berghe et al., 2015; Ahn et al., 2021) of need supportive teacher behaviors were also emphasized in the study by Ahn et al. (2019), in that a unidimensional factor structure best represented the data. These findings raise the question if this unidimensional factor structure of need supportive teacher behaviors in PE can also be transferred to other instruments. Indeed, there are clear differences in the operationalization of the constructs, which suggest a more or less pronounced distinctiveness of the dimensions with regard to the item content. An example of this is the PE-adapted version of the Learning Climate Questionnaire (Standage et al., 2005), in which item formulations such as “we feel understood by our PE teacher” or “the PE teacher shows confidence in our abilities to do well in PE” were subsumed under autonomy support instead of relatedness or competence support. A three-dimensional factor structure appears less suitable for corresponding instruments than composite or latent scores. In our opinion, an approach that attempts to investigate the independent influence of the different need supportive teacher behaviors should have a correspondingly clear conceptual distinction of the constructs. Furthermore, we extend this statement to include the construct of peer relatedness support, which potentially has a unique explanatory power on students’ intrinsic motivation and perceived competence and represents a promising area of research due to the limited recognition so far (Vasconcellos et al., 2020). Accordingly, the present study aims to examine the factorial structure of the basic psychological need support as well as the effects on intrinsic motivation and perceived competence on both the individual and class level.

Moreover, a special characteristic of the present study is the focus on changes in intrinsic motivation and perceived physical competence in individual sports. In this context, the exercise and self-esteem model (EXSEM) emphasizes that confidence in one’s abilities in specific sport-related activities can be generalized to a broader perceived physical competence (Sonstroem et al., 1994). Furthermore, this approach is intended to enable the most action-proximal and most realistic representation of PE lessons, which are largely realized in the context of specific sports. It is assumed that a positive motivational development as well as perceived competence in specific sports can be transferred to PE in general but enables to a more fine-grained resolution of the support of the students in a particular teaching series. Consequently, the following research questions are addressed:

1. To what extent does the four-factor model of the basic psychological need support, including autonomy support, competence support, teacher relatedness support and peer relatedness support, adequately represent the data at individual and class level using multilevel confirmatory factor analysis? We hypothesize that a latent factor model with four individual- and class-level dimensions will provide the best fit to the data. (H1)
2. How does the students’ perception of the basic psychological need support influence the development of intrinsic motivation in basketball at the individual and class level? We assume that all four factors are significant predictors for intrinsic motivation at both the individual and class level. With regard to supportive teacher behaviors, we assume that autonomy support in particular acts as a meaningful predictor. Furthermore, we assume that peer relatedness support explains an independent part of the variance. (H2)
3. How does the students’ perception of the basic psychological need support influence the development of perceived competence in basketball at the individual and class level? We assume that all four factors are significant predictors for perceived competence at both the individual and class level, whereby it is hypothesized that autonomy and competence support represent stronger predictors than teacher relatedness support. Furthermore, we assume that peer relatedness support explains an independent part of the variance. (H3)

## 2 Materials and methods

### 2.1 Study design and participants

Data stems from the EPiC-PE study (Messmer et al., 2022), which aims to investigate the effects of professional competencies of PE teachers on instructional quality and students´ outcomes. The analysis of the factor structure involved 1,047 students from 72 seventh- through ninth-grade classes. While for research question 1 the full data set could be used, for research question 2 and 3 we used a subsample of 735 students from 49 classes which also participated in the analysis of the longitudinal effects on intrinsic motivation and perceived competence in basketball.^1^ Secondary schools in several German-speaking Swiss cantons were contacted in order to recruit participants. Data collection took place between October 2021 and April 2022. The study involved two measurement points with a teaching series of 12 lessons between them. The completion time for the entire survey section took 15–20 min at each measurement point and was supplemented by a knowledge test. Beforehand, the students received a short explanation from their physical education teacher, who was trained for this purpose by means of a standardized written explanation. Parents were informed prior that participation was voluntary and were required to sign an informed consent form. Students were also informed that participation was voluntary. No incentives were given for participation. The teachers had to complete their own questionnaire and were present during the entire assessment. Teachers were given motor goals (e.g., “Students will be able to dribble a ball safely and shielded around obstacles”) from the swiss PE curriculum to achieve in the 12-lesson teaching series. In general, the teachers were supposed to realize their lessons in such a way that they led to the achievement of the corresponding goals. The teaching was conducted without further instruction, so that the variance in support of the basic psychological needs can be attributed as much as possible to the actual, natural performance of the teachers. Students rated perceived need support at the second measurement point, referring to the teaching series, so that the assumption seems to be more plausible, that the perceived need support is causally prior to the outcomes (Naumann et al., 2020). Intrinsic motivation and perceived competence in basketball were assessed via repeated measures directly before and after the teaching series, so that an investigation of the development becomes possible. The average class size for the sample is 14.5 students per class. The average age of students drawn from grades seven to nine is 14.5 years (SD = 1.6). Forty-seven percent of the subjects were female.

### 2.2 Measures

Intrinsic motivation for basketball was assessed by a scale consisting three items (e.g., “Basketball is fun for me”) adapted from Büchel (2019). Perceived competence in basketball was assessed by a scale also comprises three items (e.g., “I’m very good at basketball”) adapted from (Gerlach, 2008). Need supportive teacher behaviors and peer relatedness support were assessed, adapting the items from the COACTIV and Pythagoras study (Baumert et al., 2009; Rakoczy et al., 2013). Before completing the items, the students were given the following instruction: “To what extent do you agree with the following statements about your physical education class and your physical education teacher in the last teaching series?.” Perceived autonomy support contained four items (e.g., “In my PE class, I have the opportunity to explore new movements independently”), competence support also contained four items (e.g., “In my PE class, it is recognized when I accomplish something”), teacher relatedness support contained three items (e.g., “Our PE teacher takes care of the students’ problems”) and peer relatedness support contained four items (e.g., “In our PE class, I am treated as a friend by the others in the class”). All variables were completed on a Likert scale from 1 (totally disagree) to 4 (totally agree).

### 2.3 Analyses

The factorial structure was analyzed via multilevel confirmatory factor analysis (MCFA) that was specified doubly latent accordingly to the approach of Marsh et al. (2009). We compared the hypothesized four-factor structure with alternative models. In the next step, we analyzed longitudinal measurement invariance of intrinsic motivation and perceived competence to disentangle whether the observed differences in the two variables are due to a true individual difference in means or whether they are structural differences (Miyamoto et al., 2020). Three levels of measurement invariance with different numbers of model parameters were conducted, starting with configural measurement variance, followed by metric and scalar measurement invariance (Chen, 2007). Two correlated factors (T1, T2) were specified in the configural invariance model. Factor means were fixed to 0, factor variances were fixed to 1 for model identification and the co-variances and residual co-variances of the same indicators were freely estimated across the two measurement points. In the metric measurement invariance model, the factor variances were fixed to 1 for model identification at T1 and freely estimated for T2, and the factor loadings were constrained to be equal over time. Finally, the scalar measurement invariance model was tested to examine a significant comparison of means over time, in which the intercepts were additionally constrained to be equal over time. To examine the prediction of the need supportive practices on the development of intrinsic motivation and perceived competence, doubly manifest multilevel regression analyses were specified, with single manifest indicators for the scales and a manifest aggregation of the level 1 units at the class level (Marsh et al., 2009). Group mean centering (Lüdtke et al., 2009) for level 1 variables of supportive practices was used. Gender was introduced as a manifest covariate at the within level.

All models were estimated using Mplus 8.7 (Muthén and Muthén, 2017) with robust Maximum Likelihood estimation (MLR), which is robust against non-normality of item responses. Despite the categorical variables, we preferred MLR estimation over weighted least squares means and variance adjusted (WLSMV) estimation, following the practice of Aguado et al. (2015). In this context we specify at least four response options on a frequency scale (Beauducel and Herzberg, 2006) and we can use the “missing at random” (Asparouhov and Muthén, 2010) handling of missing data. Goodness-of-fit was assessed using the absolute fit indices, adhering to conventional cutoff values from Hu and Bentler (1999): standardized root mean square residual (SRMR) ≤ 0.08; root mean square error of approximation (RMSEA) ≤ 0.06; comparative fit index (CFI) and tucker-lewis index (TLI) ≥ 0.95 as well as χ^2^ /df-Ratio (Wheaton et al., 1977). The proportion of missing values per item was between 0.0 and 1.4%. Missing values were addressed using the full information maximum likelihood estimator (FIML).

## 3 Results

Descriptives (Tables 1, 2) indicate good to very good reliability of the scales. An increase in the observed mean value of intrinsic motivation and perceived competence can be found between the two measurement points, which is particularly evident in the case of perceived competence. Intraclass correlation coefficients (ICCs) as well as design effects indicate substantial dependence of clustering of the data within classes. In this context ICC1 values higher than 0.05 indicate meaningful correlations of variables between and within. ICC2 values higher than 0.60 indicate a meaningful aggregation of the individual-level data on the class level (Bliese, 2000; Chen et al., 2004). Only peer relatedness support showed ICC2 values that were slightly below the cut-off, indicating insufficient reliability of the scale at the class level.

**Table 1:** Descriptives: basic psychological need support.

Item example (1 = not true at all; 4 = exactly true)								
Factor	Items	(“In our physical education class…”; “Our physical education teacher…”)	*M*	SD	α	ω	ICC1	ICC2
Autonomy support	4	I have the opportunity to explore new movements independently.	2.93	0.65	0.82	0.82	≥0.10	≥0.62
Competence support	4	It is recognized when I accomplish something.	2.99	0.66	0.86	0.86	≥0.13	≥0.75
Teacher relatedness support	3	Takes care of the students’ problems.	3.12	0.70	0.84	0.84	≥0.17	≥0.68
Peer relatedness support	4	I feel understood by the other students in the class.	3.23	0.71	0.90	0.90	≥0.09	≥0.59

**Table 2:** Descriptives: intrinsic motivation and perceived competence.

Factor	Items	Item example (1 = not true at all; 4 = exactly true)	*M*	SD	α	ω
Intrinsic motivation T1	3	Basketball is fun for me.	2.73	0.84	0.94	0.94
Intrinsic motivation T2	3	Basketball is fun for me.	2.83	0.82	0.93	0.93
Perceived competence T1	3	I’m very good at basketball.	2.47	0.66	0.86	0.86
Perceived competence T2	3	I’m very good at basketball.	2.7	0.59	0.82	0.82

### 3.1 Research question 1: factor structure

To address research question 1, we examined the factor structure of the basic psychological need support using MCFA (Table 3). First, we tested a unidimensional solution of an overall need supportive environment, which showed a poor model fit. A model with two factors, in which need supportive teacher behaviors and peer relatedness support were specified as separate factors, showed a better, but still insufficient model fit. In the next step, we compared different models with three factors, which encompass different ways of combining individual dimensions of need supportive teacher behaviors, whereas peer relatedness support was still specified as an independent factor. These models indicated a further improvement in model fit. However, the hypothesized model with four factors showed a considerably better model fit, which was completely satisfactory in the context of conservative fit statistics at the individual level (Hu and Bentler, 1999). The SRMR between, for which it is difficult to state cut-off values as sample sizes in multilevel models often tend to have less than 200 clusters (Asparouhov and Muthén, 2018), was also in a comparatively good range at 0.11. Table 4 presents the inter-factor correlations at individual and class level with respect to the hypothesized four factor structure, ranging from *r* = 0.47 to *r* = 0.75 at individual level and *r* = 0.45 to *r* = 0.92 at class level. The overall correlational pattern is in line with expectations, being higher between conceptual closer dimensions at both levels. All factor loadings were found to be statistically significant. On the individual level, standardized factor loadings for all items ranged from λ = 0.69 to λ = 0.85, while on the class level, factor loadings ranged from λ = 0.67 to λ = 1.00. Two heywood cases appeared at the class level, in which the only minor negative residual variances were fixed to zero (Hox et al., 2017).

**Table 3:** Doubly latent multilevel confirmatory factor analysis of basic psychological need support.

	χ^2^	*df*	χ^2^/*df*	CFI	TLI	RMSEA	SRMR within	SRMR between
Hypothesized model								
4-factor model	429	179	2.39	0.97	0.96	0.04	0.03	0.11
Alternative models								
3-factor model (AS & CS as one Factor)	726	186	3.90	0.93	0.92	0.05	0.04	0.12
3-factor model (AS & TRS as one Factor)	936	186	5.03	0.90	0.89	0.06	0.05	0.14
3-factor model (CS & TRS as one Factor)	943	186	5.07	0.90	0.89	0.06	0.05	0.12
2-factor model (AS,CS,TRS as one Factor)	1,213	191	6.35	0.87	0.85	0.07	0.06	0.14
1-factor model	2,597	194	13.38	0.68	0.66	0.11	0.1	0.23

AS, autonomy support; CS, competence support; TRS, teacher relatedness support.

**Table 4:** Inter-factor correlations at individual and class level.

Factor	AS	CS	TRS	PRS
AS	1	0.75^**^	0.60^**^	0.56^**^
CS	0.92^**^	1	0.62^**^	0.57^**^
TRS	0.89^**^	0.90^**^	1	0.47^**^
PRS	0.45^**^	0.47^**^	0.52^**^	1

AS, autonomy support; CS, competence support; TRS, teacher relatedness support; PRS, peer relatedness support. *p < 0.05. **p < 0.01.

### 3.2 Research question 2 and 3: prediction of intrinsic motivation and perceived competence

#### 3.2.1 Measurement invariance

Table 5 shows the results of the longitudinal measurement invariance testing of intrinsic motivation and perceived competence. The results of the models regarding conservative fit statistics show that the models with three indicators each exhibit scalar measurement invariance. This indicates that the structure remained the same across the two measurement points and that a comparison of the mean values can be justified. Another interesting finding relates to the latent mean difference over time, which is significant for both variables (intrinsic motivation = 0.11, *p* = 0.00; perceived competence = 0.26, *p* = 0.00).

**Table 5:** Longitudinal measurement invariance of intrinsic motivation and perceived competence.

Model	χ^2^	*df*	*p*	CFI	TLI	RMSEA
Intrinsic motivation						
Configural invariance	42.05	8	0.00	0.99	0.98	0.08
Metric invariance	46.46	10	0.00	0.99	0.99	0.07
Scalar invariance	46.51	12	0.00	0.99	0.99	0.06
Difference between models	configural/metric: Δχ^2^ (Δ*df* = 2) = 4.42, *p* = 0.35; metric/scalar: Δχ^2^ (Δ*df* = 2) = 0.06, *p* = 0.97					
Perceived Competence						
Configural invariance	63.54	8	0.00	0.96	0.93	0.10
Metric invariance	68.44	10	0.00	0.96	0.95	0.09
Scalar invariance	68.59	12	0.00	0.96	0.96	0.08
Difference between models	configural/metric: Δχ2 (Δ*df* = 2) = 5.05, *p* = 0.28; metric/scalar: Δχ2 (Δ*df* = 2) = 0.15, *p* = 0.93					

#### 3.2.2 Intrinsic motivation

After the longitudinal measurement invariance was tested, we continued with the multilevel regression analyses. Table 6 shows the stepwise analysis of the inclusion of individual predictors (M1-M5) and the simultaneous consideration of all predictors in one model (M6). First of all, it can be noted that motivation at T1 is a strong predictor at both levels for motivation at T2. Furthermore, gender is also a significant predictor at the individual level. With regard to the separate integration of need support dimensions, only autonomy support and peer relatedness support can be identified as significant predictors for the development of intrinsic motivation at the individual level. Competence support and teacher relatedness support do not represent significant predictors. Only the effect of peer relatedness support also remains in Model 6 when all predictors are considered together. At the class level, in the separate integration of the need support variables, competence support, teacher relatedness support and peer relatedness support are significant predictors, whereas autonomy support represents no significant predictor (*p* < 0.067). However, due to the small number of clusters, we have also provided values at the class level at the *p* < 0.10 significance level. Regarding the simultaneous analysis of all predictors in M6, only the peer relatedness support remains significant, analogous to the individual level. The predictors in M6 explained 48% of individual-level variance and 66% of between-level variance in students’ intrinsic motivation at T2. However, need support at the individual level only explained additional 1% of variance, while at class level 11% of variance could be explained.

**Table 6:** Doubly manifest multilevel regression analysis: intrinsic motivation.

	M1		M2		M3		M4		M5		M6	
	Est.	SE	Est.	SE	Est.	SE	Est.	SE	Est.	SE	Est.	SE
Within												
Motivation, T1	0.68^*^	0.03	0.67^*^	0.03	0.66^*^	0.02	0.67^*^	0.03	0.66^*^	0.03	0.66^*^	0.03
Gender	0.13^*^	0.05	0.13^*^	0.05	0.13^*^	0.05	0.13^*^	0.05	0.13^*^	0.05	0.13^*^	0.05
Autonomy support			0.06^*^	0.03							0.03	0.04
Competence support					0.06	0.04					0.03	0.05
Teacher relatedness support							0.03	0.03			−0.02	0.04
Peer relatedness support									0.08^*^	0.03	0.07^*^	0.04
R^2^	0.47		0.48		0.48		0.48		0.48		0.48	
Between												
Motivation, T1	0.74^*^	0.07	0.69^*^	0.09	0.62^*^	0.10	0.64^*^	0.09	0.68^*^	0.08	0.59^*^	0.09
Autonomy support			0.17^a^	0.09							−0.11	0.15
Competence support					0.28^*^	0.11					0.22	0.24
Teacher relatedness support							0.26^*^	0.11			0.10	0.15
Peer relatedness support									0.29^*^	0.14	0.22^*^	0.09
R^2^	0.55		0.58		0.61		0.61		0.63		0.66	

Standardized regression coefficients; ^*^p < 0.05.

a *p* < 0.10.

#### 3.2.3 Perceived competence

With regard to the prediction of perceived competence at T2, perceived competence at T1 was a strong predictor at both levels. As for intrinsic motivation, gender was a significant predictor for the development of perceived competence at the individual level. With regard to the separate consideration of individual need support variables, all predictors except teacher relatedness support were significant at the individual level. Peer relatedness support represents again the strongest predictor. The simultaneous consideration of all predictors in one model (M6) showed, similar to intrinsic motivation, that peer relatedness support remains the only significant predictor when the other need support variables are taken into account. At the class level, all four need support dimensions were significant predictors, with autonomy and competence support being the strongest. However, when all predictors in M6 were considered simultaneously, only peer relatedness support remains significant. Also in this case, due to the small number of clusters, we have provided values at the class level at the *p* < 0.10 level, in which autonomy support acts as a significant predictor (*p* = 0.095). The predictors in M6 explained 40% of individual-level variance and 52% of between-level variance in students’ perceived competence at T2. Need supportive practices at the individual level explained additional 3% of variance, while at class level 19% of variance could be explained.

## 4 Discussion

Numerous research findings within and outside of physical education emphasize the importance of providing support for the three basic psychological needs. However, little attention has been paid to the individual and combined contributions of the different need support dimensions for positive motivational development and optimal human functioning. As Ryan and Deci (2017) note, the focus on autonomy support as a predictor of basic need satisfaction is in no way due to the idea that autonomy is more important than competence or relatedness. However, SDT postulates that autonomy support as a contextual factor is particularly significant when it comes to the active satisfaction of all individual’s needs (Ryan and Deci, 2017). Against this background, only a few studies have investigated basic psychological need support in terms of multidimensionality. Likewise, only few studies have investigated peer relatedness support in physical education (Vasconcellos et al., 2020). Finally, there are hardly any research findings to date that take the clustered data structure into account using a multilevel approach, even though the support of the three basic psychological needs are characteristics of a learning environment. Derived from these desiderata, we proposed a four-factor structure that differentiates relatedness support into teacher relatedness support and peer relatedness support in addition to autonomy support and competence support and tested this model with regard to the factorial structure and the prediction of the development of intrinsic motivation and perceived competence at individual and class level within the context of a teaching series in physical education.

Our factor analytical results support the hypothesized four-factor model and thus the assumption that autonomy support, competence support, teacher relatedness support and peer relatedness support represent separate but highly correlated dimensions at both individual and class level. Different alternative models showed a poorer model fit (ΔCFI = −0.04; ΔTLI = −0.04; ΔRMSEA = +0.01; ΔSRMR within/between = +0.01). This finding is interesting in the context of previous studies that were unable to confirm a multidimensional structure (Ahn et al., 2019). One possible explanation lies in the heterogeneity of the conceptualizations and measurements of basic psychological needs support. A systematic analysis of different operationalizations and the associated factor analytical results as well as the predictive evidence appears to be a worthwhile endeavor. Our approach corresponds to an operationalization with items that are clearly assignable. It is also interesting to note that the factorial structure also favors a four-factor solution at the class level, as a less differentiated number of factors is a common phenomenon (Dedrick and Greenbaum, 2011). Moreover, the high inter-factor correlations could indicate dependencies in the function of the individual variables (e.g., autonomy support as a prerequisite for teacher relatedness support).

With regard to the prediction of intrinsic motivation (Table 6) and perceived competence (Table 7), the results of the multilevel regression analyses were partially in line with our expectations. As assumed, the separate integration of individual dimensions of students’ shared perceptions at the class level showed that supporting the basic psychological needs resulted in a positive development of intrinsic motivation. However, autonomy support could only be shown to be a substantial predictor at the *p* < 0.10 significance level. When all predictors were considered simultaneously, only the perceived support of relatedness by peers remained significant. At the individual level, it was also shown that students who felt more connected to their peers compared to their classmates and who perceived more autonomy and competence support compared to their classmates showed a positive motivational development. However, in the simultaneous analysis of all predictors, again, only peer relatedness support remained significant.

**Table 7:** Doubly manifest multilevel regression analysis: perceived competence.

	M1		M2		M3		M4		M5		M6	
	Est.	SE	Est.	SE	Est.	SE	Est.	SE	Est.	SE	Est.	SE
Within												
Perceived Comp., T1	0.60^*^	0.04	0.58^*^	0.04	0.57^*^	0.04	0.59^*^	0.04	0.57^*^	0.04	0.56^*^	0.04
Gender	0.10^*^	0.04	0.11^*^	0.04	0.11^*^	0.04	0.11^*^	0.04	0.11^*^	0.04	0.11^*^	0.04
Autonomy support			0.08^*^	0.04							0.01	0.04
Competence support					0.11^*^	0.04					0.07	0.05
Teacher relatedness support							0.05	0.05			−0.03	0.05
Peer relatedness support									0.14^*^	0.03	0.12^*^	0.03
R^2^	0.37		0.38		0.38		0.38		0.39		0.40	
Between												
Perceived Comp., T1	0.58^*^	0.14	0.49^*^	0.14	0.46^*^	0.15	0.49^*^	0.15	0.50^*^	0.11	0.43^*^	0.14
Autonomy support			0.39^*^	0.13							0.32^a^	0.19
Competence support					0.37^*^	0.13					0.06	0.24
Teacher relatedness support							0.32^*^	0.14			−0.04	0.17
Peer relatedness support									0.30^*^	0.14	0.23^*^	0.09
R^2^	0.33		0.48		0.46		0.43		0.42		0.52	

Standardized regression coefficients; ^*^p < 0.05.

a *p* < 0.10.

Similar findings were found for the development of perceived competence. At the class level, all four support dimensions were significant predictors. However, in contrast to intrinsic motivation, autonomy support was the strongest predictor, followed by competence support and peer relatedness support, in line with our second hypothesis. In the simultaneous analysis of all predictors, peer relatedness support was again found to have a unique predictive power, whereas autonomy support was significant only at the *p* < 0.10 significance level. At the individual level, it can be basically stated that students who perceived their teacher as more autonomy- and competence-supportive compared to their classmates, showed a positive development of perceived competence. Peer relatedness support also had a positive effect at the individual level, whereas only teacher relatedness support showed no effect. These findings are in line with expectations and correspond to the assumption outlined above that autonomy and competence support can be influenced by the teacher in particular. In the simultaneous analysis of all predictors, as with intrinsic motivation, only peer relatedness support remained significant. The unique predictive power of peer relatedness support is in line with our expectations (see H2), as a different group of reference is addressed. Nevertheless, it was expected that the support of autonomy would also remain significant. Therefore, these findings could indicate a lack of incremental predictive validity of the three dimensions of supportive teacher behaviors. Although this finding is congruent with the SDT’s focus on autonomy support (Ryan and Deci, 2017) and the unidimensional modeling of supportive teacher behavior, a relatively strict separation of the three dimensions does not indicate that autonomy support is more meaningful with regard to intrinsic motivation.

These results should be viewed critically against the background of the parsimonious operationalization. Studies operationalize autonomy support in a very heterogeneous way, such as quite narrow as the merely provision of choice up to a very comprehensive multidimensional measurement (Stefanou et al., 2004; Zimmermann et al., 2020; Ahmadi et al., 2023). In our case, the instrument integrated a motivating component for autonomous work, the possibility of independent examination of the learning object, the provision of choice and openness to suggestions. In line with classification systems (Teixeira et al., 2020; Ahmadi et al., 2023), future research should critically reflect on the different operationalizations of the constructs and eventually reinterpret effects. However, in order to gain a better understanding of the mechanisms of action of the different need supportive behaviors, to design targeted interventions and to inform teacher practices, the multidimensional approach, investigating the individual contributions and the interaction of the different support dimensions in the promotion of motivational and competence-related outcomes, appears to be a worthwhile endeavor for future studies. In this context, tailoring educational strategies to meet students’ needs by understanding various dimensions of need support could significantly enhance instructional efficacy. To provide appropriate support for each student and not to embody a controlling, cold or chaotic teaching style (e.g., Van den Berghe et al., 2013; Aelterman et al., 2019), simultaneous support for all needs seems essential. One possible explanation that these potential additive effects of basic psychological needs support aren’t reflected in our data is that data originates from students’ subjective perceptions. Against this background, the items refer to the rating of the teaching or the teacher and thus show clear parallels with research on instructional quality, in which the ability of students to sufficiently differentiate between different dimensions with conceptual overlap is critically reflected (e.g., Kruse et al., 2024). For example, an affective overall attitude in the sense of a halo effect across the individual dimensions could be present (e.g., Wallace et al., 2016; Röhl and Rollett, 2021). External observation studies would represent a more objective data source in this regard and could be applied in future studies. Similar effects could be shown for student’s engagement, where it could be demonstrated that in externally rated collective behavioral engagement an additional effect of autonomy support and structure was present, whereas there were small effects and no independent explanatory power of structuring over autonomy support in relation to self-reported individual engagement (Jang et al., 2010). Further important limitations are the relatively short survey period, in which effects were examined over a period of only six weeks. An investigation over a longer period would appear to be a valuable and complementary endeavor. A larger sample would be desirable for the use of multilevel structural equation modeling of the longitudinal data so that sample and measurement error can be addressed (e.g., Marsh et al., 2009).

Furthermore, it would be worthwhile to examine differential effects as well as the associations with related constructs that could potentially be significant for the perceived support of the three basic psychological needs, such as teachers’ professional competence (Wittwer et al., 2023), teachers’ continuing professional development (Büchel et al., 2023) or the satisfaction of the psychological needs (e.g., Vasconcellos et al., 2020) - both as an independent outcome and as a mediator for the development of intrinsic motivation. Finally, the findings highlight the role of peer relatedness support, which has received little attention in research so far (e.g., Vasconcellos et al., 2020). The results provide important evidence that peer relatedness support has a significant impact on both the intrinsic motivation and perceived competence of students. This underlines the need to specifically encourage peer interactions within PE lessons in order to create a supportive learning environment that is motivating and strengthens the perception of competence.

## Data availability statement

The datasets presented in this article are not readily available because a data embargo is in place until the completion of qualification work within the context of the project by February 2025. Requests to access the datasets should be directed to the corresponding author, and from February 2025 on SWISSUbase.

## Ethics statement

Ethical approval was not required for this study in accordance with the local legislation and institutional requirements. Written informed consent for participation in this study was provided by the participants’ legal guardians/next of kin.

## Author contributions

FK: Formal analysis, Writing – original draft, Writing – review & editing. SB: Conceptualization, Writing – review & editing. CB: Conceptualization, Writing – review & editing.

## Footnotes

^1^The teaching series was carried out in the area of invasion games using basketball and handball as examples. Due to the consistently strong emphasis (>80%) on basketball, the analyses only focus on basketball.

## References

[ref1] Aelterman N. Vansteenkiste M. Haerens L. Soenens B. Fontaine J. R. J. Reeve J. (2019). Toward an integrative and fine-grained insight in motivating and demotivating teaching styles: the merits of a circumplex approach. J. Educ. Psychol. 111, 497–521. doi: 10.1037/edu0000293

[ref2] Aguado J. Luciano J. V. Cebolla A. Serrano-Blanco A. Soler J. García-Campayo J. (2015). Bifactor analysis and construct validity of the five facet mindfulness questionnaire (FFMQ) in non-clinical Spanish samples. Front. Psychol. 6:404. doi: 10.3389/fpsyg.2015.00404 25914664 PMC4390906

[ref3] Ahmadi A. Noetel M. Parker P. Ryan R. M. Ntoumanis N. Reeve J.. (2023). A classification system for teachers’ motivational behaviors recommended in self-determination theory interventions. J. Educ. Psychol. 115, 1158–1176. doi: 10.1037/edu0000783

[ref4] Ahn I. Chiu M. M. Patrick H. (2021). Connecting teacher and student motivation: student-perceived teacher need-supportive practices and student need satisfaction. Contemp. Educ. Psychol. 64:101950. doi: 10.1016/j.cedpsych.2021.101950

[ref5] Ahn I. Patrick H. Chiu M. M. Levesque-Bristol C. (2019). Measuring teacher practices that support student motivation: examining the factor structure of the teacher as social context questionnaire using multilevel factor analyses. J. Psychoeduc. Assess. 37, 743–756. doi: 10.1177/0734282918791655

[ref6] Asparouhov T. Muthén B. (2010). Weighted least squares estimation with missing data. Mplus Tech. Append. 2010:5.

[ref7] Asparouhov T. Muthén B. (2018). SRMR in Mplus. Available at: http://www.statmodel.com/download/SRMR2.pdf

[ref8] Baumert J. Blum W. Brunner M. Dubberke T. Jordan A. Klusmann U.. (2009). Professionswissen von Lehrkräften, kognitiv aktivierender Mathematikunterricht und die Entwicklung von mathematischer Kompetenz (COACTIV): Dokumentation der Erhebungsinstrumente: Max-Planck-Institut für Bildungsforschung.

[ref9] Beauducel A. Herzberg P. Y. (2006). On the performance of maximum likelihood versus means and variance adjusted weighted least squares estimation in CFA. Struct. Equ. Model. Multidiscip. J. 13, 186–203. doi: 10.1207/s15328007sem1302_2

[ref10] Behzadnia B. Adachi P. J. C. Deci E. L. Mohammadzadeh H. (2018). Associations between students’ perceptions of physical education teachers’ interpersonal styles and students’ wellness, knowledge, performance, and intentions to persist at physical activity: a self-determination theory approach. Psychol. Sport Exerc. 39, 10–19. doi: 10.1016/j.psychsport.2018.07.003

[ref11] Biddle S. J. Ciaccioni S. Thomas G. Vergeer I. (2019). Physical activity and mental health in children and adolescents: an updated review of reviews and an analysis of causality. Psychol. Sport Exerc. 42, 146–155. doi: 10.1016/j.psychsport.2018.08.011

[ref12] Bliese P. D. (2000). Within-group agreement, non-independence, and reliability: Implications for data aggregation and analysis. In Multilevel theory, research, and methods in organizations: Foundations, extensions, and new directions. eds. Klein K. J. Kozlowski S. W. J. (Jossey-Bass/Wiley), pp. 349–381.

[ref13] Büchel S. (2019). Lehrermotivation im Sportunterricht. Cham: Springer.

[ref14] Büchel S. Kruse F. Brühwiler C. (2023). Zur Bedeutung von inhaltsbezogenem Interesse und professionellem Weiterentwicklungsverhalten für das Professionswissen von Sportlehrpersonen. Schweizerische Zeitschrift Bildungswissenschaften 45, 138–150. doi: 10.24452/sjer.45.2.5

[ref15] Chatzisarantis N. L. D. Hagger M. S. (2009). Effects of an intervention based on self-determination theory on self-reported leisure-time physical activity participation. Psychol. Health 24, 29–48. doi: 10.1080/08870440701809533, PMID: 20186638

[ref16] Chen F. F. (2007). Sensitivity of goodness of fit indexes to lack of measurement invariance. Struct. Equ. Model. Multidiscip. J. 14, 464–504. doi: 10.1080/10705510701301834

[ref17] Chen G. Mathieu J. E. Bliese P. D. (2004). A framework for multi-level construct validation. In Research in multilevel issues: Multilevel issues in organizational behavior and processes. eds. Yammarino F. J. Dansereau F., Vol. 3. (Elsevier), pp. 273–303.

[ref18] Cheon S. H. Reeve J. (2013). Do the benefits from autonomy-supportive PE teacher training programs endure?: a one-year follow-up investigation. Psychol. Sport Exerc. 14, 508–518. doi: 10.1016/j.psychsport.2013.02.002

[ref19] Cheon S. H. Reeve J. Moon I. S. (2012). Experimentally based, longitudinally designed, teacher-focused intervention to help physical education teachers be more autonomy supportive toward their students. J. Sport Exerc. Psychol. 34, 365–396. doi: 10.1123/jsep.34.3.365, PMID: 22691399

[ref20] Corder K. Winpenny E. Love R. Brown H. E. White M. Sluijs E. Van. (2019). Change in physical activity from adolescence to early adulthood: a systematic review and meta-analysis of longitudinal cohort studies. Br. J. Sports Med., 53, 496–503. doi: 10.1136/bjsports-2016-097330, PMID: 28739834 PMC6250429

[ref21] Cox A. Duncheon N. McDavid L. (2009). Peers and teachers as sources of relatedness perceptions, motivation, and affective responses in physical education. Res. Q. Exerc. Sport 80, 765–773. doi: 10.1080/02701367.2009.10599618, PMID: 20025118

[ref22] Cox A. E. Ullrich-French S. Madonia J. Witty K. (2011). Social physique anxiety in physical education: social contextual factors and links to motivation and behavior. Psychol. Sport Exerc. 12, 555–562. doi: 10.1016/j.psychsport.2011.05.001

[ref23] Crane J. Temple V. (2015). A systematic review of dropout from organized sport among children and youth. Eur. Phys. Educ. Rev. 21, 114–131. doi: 10.1177/1356336X14555294

[ref24] Dedrick R. F. Greenbaum P. E. (2011). Multilevel confirmatory factor analysis of a scale measuring interagency collaboration of children’s mental health agencies. J. Emot. Behav. Disord. 19, 27–40. doi: 10.1177/1063426610365879, PMID: 21528103 PMC3082154

[ref25] Dumith S. C. Gigante D. P. Domingues M. R. Kohl H. W. (2011). Physical activity change during adolescence: a systematic review and a pooled analysis. Int. J. Epidemiol. 40, 685–698. doi: 10.1093/ije/dyq272, PMID: 21245072

[ref26] Gairns F. Whipp P. R. Jackson B. (2015). Relational perceptions in high school physical education: teacher- and peer-related predictors of female students’ motivation, behavioral engagement, and social anxiety. Front. Psychol. 6:850. doi: 10.3389/fpsyg.2015.00850, PMID: 26157404 PMC4475790

[ref27] Gerlach E. (2008). Sportengagement und Persönlichkeitsentwicklung: Eine längsschnittliche Analyse der Bedeutung sozialer Faktoren für das Selbstkonzept von Heranwachsenden: Meyer & Meyer Verlag.

[ref28] Hox J. Moerbeek M. Van de Schoot R. (2017). Multilevel analysis: Techniques and applications, third edition. London: Routledge.

[ref29] Hu L. Bentler P. M. (1999). Cutoff criteria for fit indexes in covariance structure analysis: conventional criteria versus new alternatives. Struct. Equ. Model. 6, 1–55. doi: 10.1080/10705519909540118

[ref30] Huang F. L. Cornell D. G. (2016). Using multilevel factor analysis with clustered data: investigating the factor structure of the positive values scale. J. Psychoeduc. Assess. 34, 3–14. doi: 10.1177/0734282915570278

[ref31] Jang H. Reeve J. Deci E. L. (2010). Engaging students in learning activities: it is not autonomy support or structure but autonomy support and structure. J. Educ. Psychol. 102, 588–600. doi: 10.1037/a0019682

[ref32] Jang H. Reeve J. Ryan R. M. Kim A. (2009). Can self-determination theory explain what underlies the productive, satisfying learning experiences of collectivistically oriented Korean students? J. Educ. Psychol. 101, 644–661. doi: 10.1037/a0014241

[ref33] Janssen I. LeBlanc A. G. (2010). Systematic review of the health benefits of physical activity and fitness in school-aged children and youth. Int. J. Behav. Nutr. Phys. Act. 7, 40–16. doi: 10.1186/1479-5868-7-40 20459784 PMC2885312

[ref34] Koka A. (2014). The relative roles of teachers and peers on students’ motivation in physical education and its relationship to self-esteem and health-related quality of life. Int. J. Sport Psychol. 45, 187–213. doi: 10.7352/IJSP2014.45.187

[ref35] Kruse F. Büchel S. Brühwiler C. (2024). Dimensionality of instructional quality in physical education. Obtaining individual and aggregated student’s perceptions using Bifactor exploratory structural equation modeling and multilevel confirmatory factor analysis. Front. Psychol. 10.3389/fpsyg.2024.1370407 PMC11367638 39224697

[ref9002] Ladwig M. A. Vazou S. Ekkekakis P. (2018). “My best memory is when I was done with it”: PE memories are associated with adult sedentary behavior. Transl. J. Am. Coll. Sports Med., 3, 119–129.

[ref36] Leo F. M. López-Gajardo M. A. Rodríguez-González P. Pulido J. J. Fernández-Río J. (2023). How class cohesion and teachers’ relatedness supportive/thwarting style relate to students’ relatedness, motivation, and positive and negative outcomes in physical education. Psychol. Sport Exerc. 65:102360. doi: 10.1016/j.psychsport.2022.102360, PMID: 37665833

[ref37] Lindahl J. Stenling A. Lindwall M. Colliander C. (2015). Trends and knowledge base in sport and exercise psychology research: a bibliometric review study. Int. Rev. Sport Exerc. Psychol. 8, 71–94. doi: 10.1080/1750984X.2015.1019540

[ref38] Lonsdale C. Lester A. Owen K. B. White R. L. Peralta L. Kirwan M.. (2019). An internet-supported school physical activity intervention in low socioeconomic status communities: results from the activity and motivation in physical education (AMPED) cluster randomised controlled trial. Br. J. Sports Med. 53, 341–347. doi: 10.1136/bjsports-2017-097904, PMID: 28993404

[ref39] Lüdtke O. Robitzsch A. Trautwein U. Kunter M. (2009). Assessing the impact of learning environments: how to use student ratings of classroom or school characteristics in multilevel modeling. Contemp. Educ. Psychol. 34, 120–131. doi: 10.1016/j.cedpsych.2008.12.001

[ref40] Marsh H. W. Lüdtke O. Robitzsch A. Trautwein U. Asparouhov T. Muthén B.. (2009). Doubly-latent models of school contextual effects: integrating multilevel and structural equation approaches to control measurement and sampling error. Multivar. Behav. Res. 44, 764–802. doi: 10.1080/00273170903333665, PMID: 26801796

[ref9011] Messmer R. Brühwiler C. Gogoll A. Büchel S. Vogler J. Kruse F.. (2022). “Wissen und Können bei Lehrpersonen und Lernenden im Sportunterricht. Zum Design und zur Modellierung von Schüler*innen und Lehrer*innenkompetenzen,” Narrative zwischen Wissen und Können. Aktuelle Befunde aus Sportdidaktik- und Pädagogik. Academia. eds. R. Messmer and C. Krieger (Hrsg.). doi: 10.5771/9783985720118-209

[ref41] Miyamoto A. Murayama K. Lechner C. M. (2020). The developmental trajectory of intrinsic reading motivation: measurement invariance, group variations, and implications for reading proficiency. Contemp. Educ. Psychol. 63:101921. doi: 10.1016/j.cedpsych.2020.101921

[ref42] Muthén B. Muthén L. (2017). “Mplus” in Handbook of item response theory. ed. Linden W. J. (Boca Raton, FL: Chapman and Hall/CRC), 507–518.

[ref43] Naumann A. Kuger S. Köhler C. Hochweber J. (2020). Conceptual and methodological challenges in detecting the effectiveness of learning and teaching. Zeitschrift für Pädagogik, 66, 179–196.

[ref44] Ntoumanis N. Standage M. (2009). Motivation in physical education classes: a self-determination theory perspective. Theory Res. Educ. 7, 194–202. doi: 10.1177/1477878509104324

[ref45] Poitras V. J. Gray C. E. Borghese M. M. Carson V. Chaput J.-P. Janssen I.. (2016). Systematic review of the relationships between objectively measured physical activity and health indicators in school-aged children and youth. Appl. Physiol. Nutr. Metab. 41, S197–S239. doi: 10.1139/apnm-2015-0663 27306431

[ref46] Rakoczy K. Buff A. Lipowsky F. (2013). “Wahrgenommene Autonomieunterstützung—Schüler [Fragebogenskala: Version 1.0]” in Unterrichtsqualität und mathematisches Verständnis in verschiedenen Unterrichtskulturen—Fragebogenerhebung Abschlussbefragung (Pythagoras). DIPF.

[ref47] Röhl S. Rollett W. (2021). Student perceptions of teaching quality: dimensionality and halo effects. In Student Feedback on Teaching in Schools. Eds. Rollett W. Bijlsma H. Röhl S. (Springer), pp. 31–45.

[ref48] Roth G. Assor A. Kanat-Maymon Y. Kaplan H. (2007). Autonomous motivation for teaching: how self-determined teaching may lead to self-determined learning. J. Educ. Psychol. 99, 761–774. doi: 10.1037/0022-0663.99.4.761

[ref49] Ryan R. M. Deci E. L. (2017). Self-determination theory: basic psychological needs in motivation, development, and wellness. New York: Guilford Press.

[ref50] Sanchez-Oliva D. Sanchez-Miguel P. A. Leo F. M. Kinnafick F.-E. García-Calvo T. (2014). Physical education lessons and physical activity intentions within Spanish secondary schools: a self-determination perspective. J. Teach. Phys. Educ. 33, 232–249. doi: 10.1123/jtpe.2013-0043

[ref51] Shen B. (2014). Outside-school physical activity participation and motivation in physical education. Br. J. Educ. Psychol. 84, 40–57. doi: 10.1111/bjep.12004, PMID: 24547753

[ref52] Sonstroem R. J. Harlow L. L. Josephs L. (1994). Exercise and self-esteem: validity of model expansion and exercise associations. J. Sport Exerc. Psychol. 16, 29–42. doi: 10.1123/jsep.16.1.29

[ref53] Sparks C. Lonsdale C. Dimmock J. Jackson B. (2017). An intervention to improve teachers’ interpersonally involving instructional practices in high school physical education: implications for student relatedness support and in-class experiences. J. Sport Exerc. Psychol. 39, 120–133. doi: 10.1123/jsep.2016-0198, PMID: 28787252

[ref54] Standage M. Duda J. L. Ntoumanis N. (2005). A test of self-determination theory in school physical education. Br. J. Educ. Psychol. 75, 411–433. doi: 10.1348/000709904X22359 16238874

[ref55] Stefanou C. R. Perencevich K. C. DiCintio M. Turner J. C. (2004). Supporting autonomy in the classroom: ways teachers encourage student decision making and ownership. Educ. Psychol. 39, 97–110. doi: 10.1207/s15326985ep3902_2

[ref56] Stodden D. F. Goodway J. D. Langendorfer S. J. Roberton M. A. Rudisill M. E. Garcia C.. (2008). A developmental perspective on the role of motor skill competence in physical activity: an emergent relationship. Quest 60, 290–306. doi: 10.1080/00336297.2008.10483582

[ref57] Su Y.-L. Reeve J. (2011). A Meta-analysis of the effectiveness of intervention programs designed to support autonomy. Educ. Psychol. Rev. 23, 159–188. doi: 10.1007/s10648-010-9142-7

[ref58] Taylor I. M. Ntoumanis N. Standage M. Spray C. M. (2010). Motivational predictors of physical education students’ effort, exercise intentions, and leisure-time physical activity: a multilevel linear growth analysis. J. Sport Exerc. Psychol. 32, 99–120. doi: 10.1123/jsep.32.1.99, PMID: 20167954

[ref59] Teixeira P. J. Marques M. M. Silva M. N. Brunet J. Duda J. L. Haerens L.. (2020). A classification of motivation and behavior change techniques used in self-determination theory-based interventions in health contexts. Motiv. Sci. 6, 438–455. doi: 10.1037/mot0000172

[ref60] Telama R. (2009). Tracking of physical activity from childhood to adulthood: a review. Obes. Facts 2, 187–195. doi: 10.1159/000222244 20054224 PMC6516203

[ref61] Van den Berghe L. Soenens B. Vansteenkiste M. Aelterman N. Cardon G. Tallir I. B.. (2013). Observed need-supportive and need-thwarting teaching behavior in physical education: do teachers’ motivational orientations matter? Psychol. Sport Exerc. 14, 650–661. doi: 10.1016/j.psychsport.2013.04.006

[ref62] Van Den Berghe L. Tallir I. B. Cardon G. Aelterman N. Haerens L. (2015). Student (dis)engagement and need-supportive teaching behavior: a multi-informant and multilevel approach. J. Sport Exerc. Psychol. 37, 353–366. doi: 10.1123/jsep.2014-0150, PMID: 26442767

[ref63] Vasconcellos D. Parker P. D. Hilland T. Cinelli R. Owen K. B. Kapsal N.. (2020). Self-determination theory applied to physical education: a systematic review and meta-analysis. J. Educ. Psychol. 112, 1444–1469. doi: 10.1037/edu0000420

[ref64] Wallace T. L. Kelcey B. Ruzek E. (2016). What can student perception surveys tell us about teaching? Empirically testing the underlying structure of the tripod student perception survey. Am. Educ. Res. J. 53, 1834–1868. doi: 10.3102/0002831216671864

[ref65] Wheaton B. Muthen B. Alwin D. F. Summers G. F. (1977). Assessing reliability and stability in panel models. Sociol. Methodol. 8, 84–136. doi: 10.2307/270754

[ref66] White R. L. Bennie A. Vasconcellos D. Cinelli R. Hilland T. Owen K. B.. (2021). Self-determination theory in physical education: a systematic review of qualitative studies. Teach. Teach. Educ. 99:103247. doi: 10.1016/j.tate.2020.103247

[ref67] Whooten R. Kerem L. Stanley T. (2019). Physical activity in adolescents and children and relationship to metabolic health. Curr. Opin. Endocrinol. Diabetes Obes. 26, 25–31. doi: 10.1097/MED.0000000000000455, PMID: 30507695 PMC6522241

[ref68] Wittwer M. Messmer R. Büchel S. (2023). Fachspezifisches professionelles Wissen und Können von Sportlehrpersonen. *Swiss*. J. Educ. Res. 45:2. doi: 10.24452/sjer.45.2.4

[ref69] Zimmermann J. Tilga H. Bachner J. Demetriou Y. (2020). The German multi-dimensional perceived autonomy support scale for physical education: adaption and validation in a sample of lower track secondary school students. Int. J. Environ. Res. Public Health 17:19. doi: 10.3390/ijerph17197353, PMID: 33050116 PMC7579324

